# Assessment of mortality post-ICU associated to the intensive care unit length of stay

**DOI:** 10.1186/2197-425X-3-S1-A968

**Published:** 2015-10-01

**Authors:** F Gómez Triana, P Carcelén Rodríguez, ZE Aray Delpino, D Monge Donaire, TL Álvarez Pérez, FC Tarancón Maján, SM Cortés Díaz, A Marcos Gutiérrez, AC Caballero Zirena

**Affiliations:** Virgen de la Concha Hospital, Zamora, Spain

## Introduction

Patients admitted to the ICU may require longer stays, which in turn can influence the evolution of their disease. This may be a determining factor of their overall prognosis and result in modifications to previously determined morbidity and mortality.

## Objective

To establish prolonged effects of mortality following patients with prolonged stays in the ICU once they are transferred to the general ward, and identify possible reasons associated with this mortality.

## Methods

This is a retrospective study of ICU patients during 2013 at the Virgen de la Concha Hospital (Zamora, Spain). 445 patients admitted during the study period were identified as having a prolonged ICU stay defined as equal to, or greater than 10 days. Patients were evaluated following transfer from the ICU to the general ward until death or discharge. Epidemiological data, diagnosis at admission and estimation of risk of death, were assessed by APACHE II scores.

## Results

The overall ICU mortality rate was 12.58% (IC 95%: 9,39-15,7), that is 56 of the 445 patients that were included during the study period. 53 patients had a prolonged stay (11,9%) ; 10 of which expired during their stay in the ICU (18%). The remaining 43 were discharged to the general ward. Of these 43 ward transfers, 11 (25%) expired during their stay on the ward (IC 95%: 11,3-39,7) and the remaining 32 were discharged to home. The expected deaths were 28% and the observed deaths 25% (Standardized mortality ratio of 0.89; 95% CI, 0.39 to 1,38). The average stay in the general ward post-ICU was 17.74 ± 23.98 days.

## Conclusions

Prolonged stay in the ICU is a determining factor that influences patients mortality following their transfer to the general ward. The high mortality can be predicted on the APACHE II system applied to our patient population. As a result, the APACHE II system may assist the clinician in determining which patients are at increased risk of mortality, and thus devise ways to circumvent such events in the patients with prolonged ICU stays.
